# Drug Repositioning for Hand, Foot, and Mouth Disease

**DOI:** 10.3390/v15010075

**Published:** 2022-12-27

**Authors:** Ran Yan, Jiahao He, Ge Liu, Jianfeng Zhong, Jiapeng Xu, Kai Zheng, Zhe Ren, Zhendan He, Qinchang Zhu

**Affiliations:** 1 School of Pharmaceutical Sciences, Shenzhen University, Shenzhen 518060, China; 2 College of Pharmacy, Shenzhen Technology University, Shenzhen 518118, China; 3 Institute of Biomedicine, College of Life Science and Technology, Jinan University, Guangzhou 510632, China; 4 National Engineering Research Center of Genetic Medicine, Guangzhou 510632, China

**Keywords:** drug repositioning, drug repurposing, HFMD, enteroviruses

## Abstract

Hand, foot, and mouth disease (HFMD) is a highly contagious disease in children caused by a group of enteroviruses. HFMD currently presents a major threat to infants and young children because of a lack of antiviral drugs in clinical practice. Drug repositioning is an attractive drug discovery strategy aimed at identifying and developing new drugs for diseases. Notably, repositioning of well-characterized therapeutics, including either approved or investigational drugs, is becoming a potential strategy to identify new treatments for virus infections. Various types of drugs, including antibacterial, cardiovascular, and anticancer agents, have been studied in relation to their therapeutic potential to treat HFMD. In this review, we summarize the major outbreaks of HFMD and the progress in drug repositioning to treat this disease. We also discuss the structural features and mode of action of these repositioned drugs and highlight the opportunities and challenges of drug repositioning for HFMD.

## 1. Introduction

Hand, foot, and mouth disease (HFMD) is caused by a group of enteroviruses (EVs) and was first described in 1948 [1]. It mostly occurs in children under 5 years of age and usually manifests as herpes-like lesions on the palms of the hands and soles of the feet, as well as in the mouth. However, a small number of infected children suffer from myocarditis, pulmonary edema, sterile meningoencephalitis [2], and severe neurologic and cardiorespiratory problems, potentially resulting in death, which have all also been associated with HFMD.

Pathogenic EVs belong to a family of small RNA viruses, including poliovirus, coxsackievirus (CV), and enteric cytopathic human orphan virus (echovirus). EVs isolated after the 67 types of the above-mentioned 3 groups of EVs were named according to the number of enteroviruses with a system of consecutive numbers, that is, EVs 68, 69, 70, 71, 72, etc. There are more than 20 EVs that can cause HFMD, with Group A types responsible for >90% of cases. The main EVs responsible for HFMD include CV (Group A types 4, 5, 6, 7, 9, 10, and 16 and Group B types 1, 2, 3, and 5), EV-A71 (EV71), and some serotypes of echovirus. Although CVA16 and EV71 are the most common causes of HFMD, CVA6 and CVA10 have partially replaced these as the main pathogens associated with HFMD in recent years in some places [3].

There is currently no specific drug for the treatment of HFMD, and drugs are usually only used to treat the symptoms rather than eradicate the cause. Three capsid inhibitors have been researched in clinical trials, including pleconaril, vapendavir (BTA798), and pocapavir (V073), but none of them has been approved by FDA. Pleconaril was developed to work against viruses in the picornaviridae family, including enterovirus and rhinovirus. However, due to the fact that it has been found to induce CYP3A enzyme activity, pleconaril has not gained approval by the FDA. V-073, a more recently developed capsid inhibitor, has also been demonstrated to have potent activity against polio virus and nonpolio enterovirues. BTA-798 is a pirodavir-analog with significant activity against the A and B groups of human rhinoviruses as well as enterovirues. It is currently being developed for rhinovirus infections in high risk hosts and asthmatic subjects. Interferon (IFN) α spray and intravenous infusion of ribavirin have demonstrated some efficacy in the early stage of HFMD, but their adverse reactions and side effects may be problematic [4]. IFN-α has broad-spectrum anti-viral, anti-tumor, and immunoregulatory functions. After binding to cell surface receptors, it induces cells to produce a variety of anti-viral proteins, thereby inhibiting virus replication in the cell, inhibiting the virus, and promoting recovery. Ribavirin also has a wide range of antiviral effects. It is phosphorylated after entering the cell, competitively inhibiting the synthesis of viral guanosine triphosphate and inhibiting viral mRNA synthesis. However, the effects of ribavirin on host–cell nucleic acid synthesis mean that it exhibits low selectivity, and it has also been associated with side effects such as anemia and reproductive toxicity [5]. There is thus an urgent need for safe and effective antiviral drugs to treat HFMD.

The Nobel laureate James Black said that “the most fruitful basis of the discovery of a new drug is to start with an old drug”, and, accordingly, drug repurposing/repositioning represents an attractive drug discovery strategy. Drug repositioning reduces the research and development time, as in the case of remdesivir, which was approved for the treatment of SARS-CoV-2 about 1 year after the outbreak of the pandemic [6]. Drug repositioning also reduces research and development costs, which may help pharmaceutical companies turn losses into profit and let a previously failed drug regain a new market, such as sildenafil [7]. Here, we summarize the major outbreaks of HFMD and the progress of drug repositioning for the treatment of HFMD. We also discuss the structural features and modes of action of repositioned drugs and highlight the opportunities and challenges of drug repositioning for HFMD.

## 2. Major Outbreaks of HFMD

The prevalence of HFMD shows no obvious regionality. Meteorological factors such as high temperature and humidity have been associated with HFMD susceptibility, and although onset can occur throughout the year, it is more common in summer and autumn and less common in winter. More than 90% of cases have been diagnosed in children <5 years of age, indicating age-related susceptibility to and severity of HFMD [8]. Collective infections may occur in kindergartens, nurseries, and family clusters during an epidemic. Highly contagious EVs are associated with a large proportion of hidden infections, complex transmission routes, and rapid transmission. They can cause large-scale epidemics in a short period of time, which are difficult to control.

An epidemic disease in children characterized by skin rashes and ulcers on the hands, feet, and mouth was first reported in Toronto, Canada in 1957. CVA16 was subsequently isolated from feces and throat swabs of patients with HFMD in 1958, and an outbreak of HFMD occurred in Birmingham, England in 1959. Flewett christened this new disease “hand, foot, and mouth disease”. EV71 was first isolated from children in California in 1969 and identified in 1974. EV71 infection has been responsible for HFMD epidemics since 1997, with fatal cases being especially frequent in the Asia–Pacific region. EV71 infection was first discovered in Marseille, France in 2009, and genetic sequencing showed that the responsible virus strain had 97% homology to the EV71 virus strain isolated in Singapore in 2008. However, EV71 epidemics in the Asia–Pacific region differ from those in Europe, with cases in Asia being widespread and often serious, compared with no major outbreaks in Europe. EV71 and CVA16 are the two main pathogens responsible for HFMD; however, the symptoms caused by CVA16 are generally mild, while EV71 may be fatal. Moreover, CVA16 and EV71 produce alternate epidemics. For example, there was an outbreak of HFMD in China in 2008, centered around Guangzhou, in which EV71 was the dominant virus, while CVA16 caused most cases in 2009, and EV71 re-dominated the epidemic by 2010. The causative pathogens of HFMD were subsequently identified as CVA16 and EV71, as well as other enteroviruses, such as echovirus, CV A groups 4–7, 9, and 10 and B groups 1–3, and 5. The global sequence of CVB3 shows a clustering trend specific to geographic regions. For example, the CVB3 epidemic in Hong Kong in 2008 led to a large number of patients with aseptic meningitis, and CVB3, isolated in Hong Kong, was found to interact with isolates from Fuyang, China. CVA6 was first discovered in Finland in 2008, and HFMD caused by CVA6 infection was subsequently discovered in Europe; however, HFMD infections in Europe prior to 2008 were rarely caused by CVA6. Other less-common causes of HFMD, including CVA4, CVB1, CVB5, and CVA10, are nonetheless worthy of attention, especially CVA10. HFMD caused by CVA10 can be more serious than that caused by EV71. The time periods and numbers of infections of major HFMD outbreaks caused by different enterovirus subtypes in Asian and non-Asian regions are shown in Table 1.

## 3. Drug Repositioning for HFMD

EV71 is highly infectious to the central nervous system, resulting in a series of characteristic clinical symptoms including encephalitis, aseptic meningitis, acute flaccid paralysis, herpangina, acute hemorrhagic conjunctivitis, myoclonic jerks, headache, fever, and vomiting, among which HFMD and herpangina are the most common. Most EV infections are generally asymptomatic (approximately 50–80%) or produce mild, cold-like symptoms, and affected patients recover naturally and produce antibodies. However, severely ill patients infected with EVs are at high risk of dying from cardiopulmonary failure and extensive brain stem injury. EV71 mostly affects the nervous system in children <5 years old, with the highest incidence in children aged 1–2 years. Oral ingestion of the shed virus from the gastrointestinal or upper respiratory tract of infected hosts or via vesicle fluid or oral secretions are the major means by which the virus spreads. After ingestion, the virus replicates in the lymphoid tissue of the lower intestine and the pharynx and spreads to the regional lymph nodes and then multiple organs, including the central nervous system. EV71 mainly causes neurological effects by inducing inflammation in the CNS, but not in other organs. Viral protease 3C has been shown to inhibit innate immune involvement in multiple pathological processes of EV71 by suppressing type I interferon responses. Several clinical observations provide clues that some inflammatory mediators, including cytokines and chemokines, play an important role in the pathogenesis of EV71-induced encephalitis and other complications. Despite the potentially serious effects of EV71 infection, there are currently no specific antiviral drugs, and there is thus an urgent need to identify suitable drugs. Drug repositioning provides a potential means of achieving this goal. Various drugs have been investigated for their repurposing use for HFMD, most of which are currently at the preclinical stages (Table 2).

### 3.1. Repositioning of Antifungal Drugs

**Figure 1:**
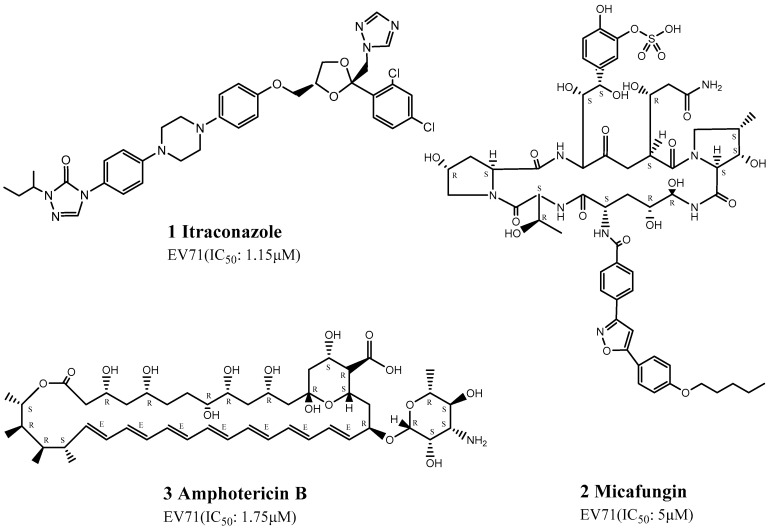
Structures of antifungal drugs.

Gao et al., Xu et al., and Chonsaeng et al. recently considered the repositioning of antifungal drugs and showed that itraconazole (ITZ) (1), micafungin (2), and amphotericin B (3) had potential pharmacological activities against EV71 infection (Figure 1). ITZ was detected by high-throughput screening assay [59] of 1280 clinical compounds in the FDA-approved drug library to detect potentially effective drugs, among which ITZ demonstrated good pharmacokinetics and safety [59]. ITZ is a new-generation triazole and is a highly-efficient, broad-spectrum antifungal drug mainly used to treat systemic infections caused by deep fungi. It can be combined with the fungal cytochrome P450 isozyme to inhibit the synthesis of ergosterol. ITZ has also been reported to act as a broad-spectrum enterovirus inhibitor, with an IC50 of 1.15 μM. Mutation of the 3A protein can cause ITZ resistance in EV71, which strongly inhibits viral RNA replication or polyprotein processing [59].

In addition, Chonsaeng et al. identified micafungin among 968 FDA-approved drugs. Micafungin is an echinocandin antifungal drug from Coleophoma empetri obtained by chemical synthesis, which shows good inhibitory activity against Candida spp., including Candida albicans, as well as in vitro inhibitory activity against Aspergillus. The researchers speculated that micafungin might target any step in early viral infection, with an EC50 of 2–10 μM. Although the mechanism of action of micafungin against EV71 infection remains unclear, it does not appear to involve internal ribosome entry site (IRES)-dependent translation or polyprotein processing involving 3Cpro, 2C, and 3A proteins [60].

Amphotericin B was found to be a potentially useful drug by Xu et al. on the basis of combination therapy used to treat co-infection by fungi and viruses [61]. Amphotericin B is a polyene antifungal drug used for systemic infections or infections of internal organs caused by Cryptococcus and Aspergillus. Amphotericin B can effectively inhibit the production of EV71, with an EC50 of 1.75 μM in Rhabdomyosarcoma (RD) cells and 0.32 μM in HEK293 cells. Amphotericin B was shown to impair the binding and internalization of EV71 virus to host cells, using western blotting, quantitative real-time polymerase chain reaction, and virus-binding assay [61].

### 3.2. Repositioning of Antibacterial Drugs

CVA16 belongs to the enterovirus genus of the Picornaviridae family and is one of the main pathogens of HFMD. CVA16 has recently been shown to cause secondary infections of the brain, lung, and heart and may even lead to fatal complications such as pneumonia, myocarditis, and refractory shock.

**Figure 2:**
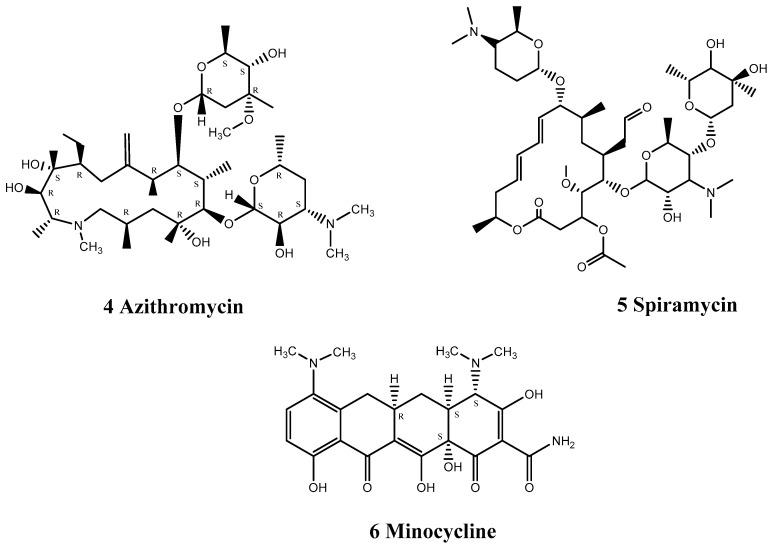
Structures of antibacterial drugs.

Zeng et al. discovered that the clinically marketed antibiotic azithromycin (4) could be used to treat EV71 and CVA16 infections (Figure 2). Azithromycin is mainly used to treat respiratory tract, skin soft tissue, and urogenital system infections but has also demonstrated antiviral activity against EV71. Azithromycin significantly reduced EV71 RNA and protein levels and probably acted by interfering with viral RNA replication [62]. Notably, azithromycin has been clinically proven to be safe in pregnant women, newborns, and young children and may thus be particularly useful, given that HFMD mostly occurs in children under 5 years of age. Notably, the combination of azithromycin with the macrolide antibiotic spiramycin (SPM) (5) inhibited the replication of EV71 and CVA16 (Figure 2), thereby exerting antiviral effects [62]. Their antiviral mechanisms appear to be similar, and EV71 mutant strains resistant to SPM show similar resistance to azithromycin. Although the specific mechanisms of these drugs are unknown, they are likely to produce antiviral effects by inhibiting virus RNA replication.

Liao et al. found that the antibiotic minocycline (6) had anti-inflammatory and immunomodulatory properties in infectious and inflammatory neurological disease models (Figure 2). They, therefore, carried out a series of in vitro and in vivo experiments to explore its effect on EV71 infection. Minocycline is a broad-spectrum antibacterial tetracycline antibiotic that can be combined with tRNA to achieve bacteriostatic effects and has the strongest antibacterial effect among tetracycline antibiotics. Unlike milrinone (drug 19), which is mainly used to treat complications of EV71 infection with no obvious anti-viral effect [63], minocycline reduced virus replication, specifically VP0 and VP2, while double-dose treatment suppressed cytokine production and viral protein expression in EV71-infected THP-1 cells [64].

### 3.3. Repositioning of Cardiovascular Drugs

CVB3 is one of the most important causative factors of viral myocarditis, accounting for 50% of cases. CVBs viruses can directly damage host cells, leading to an inflammatory host response to viral infection, resulting in target organ tissue damage and dysfunction. CVBs can harness different host cell processes including kinases, host cell-killing and cell-eating machineries, matrix metalloproteinases, and miRNAs to promote disease. Although recent improvements in treatment methods have significantly improved disease outcomes, some patients still have poor treatment responses and relapse. The incidence and mortality of CVB3-related viral myocarditis are increasing year by year. The clinical symptoms of the disease vary in severity, ranging from no obvious symptoms to shortness of breath, fatigue, chest tightness, cardiomyopathy, and even congestive heart failure and cardiogenic shock. Unfortunately, there is still no effective treatment for this disease. However, some FDA-approved drugs have been repurposed for viral myocarditis caused by CVB3 infection. These drugs have different mechanisms of action and different therapeutic effects against viral myocarditis. Since these drugs have already passed time-consuming and laborious clinical trials and have been shown to be safe, the potential repurposing of these drugs for CVB3 infection or viral myocarditis may save unnecessary costs and accelerate the research and development time.

**Figure 3:**
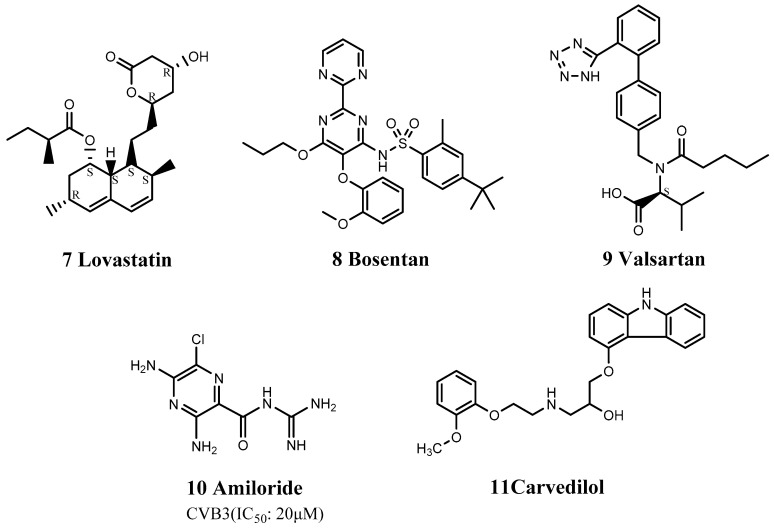
Structures of cardiovascular drugs.

Lovastatin (7), bosentan (8), and valsartan (9) inhibit the replication of CVB3 by down-regulating the expression of coxsackie-adenovirus receptor (CAR) mRNA and protein (Figure 3). CVB3 requires the human CAR to infect the myocardium. Lovastatin is a lipid-lowering drug, mainly used for the treatment of hypercholesterolemia, especially in patients with elevated low-density lipoprotein (type II). Bosentan is a specific and competitive low-molecular weight dual endothelin receptor blocker, mainly used for the treatment of pulmonary hypertension. Valsartan is an angiotensin II receptor antagonist that selectively blocks the binding of angiotensin II and angiotensin II type 1 receptor receptors, thereby inhibiting vasoconstriction and aldosterone release, resulting in a hypotensive effect. These three drugs, with completely different pharmacological effects, play important roles in inhibiting the replication of CVB3 through a similar mechanism. Werner et al. found that lovastatin decreased CAR mRNA and protein expression levels by up to 80% and 19% (*p* < 0.01), respectively [65], in a concentration-dependent manner. In contrast, bosentan drastically decreased CAR mRNA levels in HeLa cells and human umbilical vein endothelial cells (HUVECs) by 80.2% (±4.6%) and by 66.3% (±12.6%), respectively [66], while valsartan also reduced CAR mRNA in HUVECs and HeLa cells by up to 68.1% (±8.7%) and 51.4% (±8.8%), respectively [66].

Assuming that CVB3 enters the host cell through the CAR, Gazina et al. discovered that amiloride inhibited the enzymatic activity of CVB3 3Dpol in vitro [67], affecting RNA elongation. Amiloride (10) is mainly used to treat liver cirrhosis, edema, and primary aldosteronism (Figure 3). It has strong sodium-excretion and potassium-sparing diuretic effects and is mainly used to treat mild to moderate hypertension. Amiloride can block various ion channels, including the Na+/H+ exchanger, acid-sensitive ion channels, Na+/Ca^2+^ exchanger, voltage-gated Na+ channels, and Ca^2+^ channels. Moreover, Amiloride can act as a competitive inhibitor, competing with incoming nucleoside triphosphates and Mg^2+^, resulting in inhibition of CVB3 RNA replication [67].

β-adrenoreceptor agonists can activate the p38 mitogen-activated protein kinase (MAPK) pathway, leading to the expression of proinflammatory cytokines, further triggering inflammation and apoptosis. Carvedilol (11) is a β-adrenoreceptor antagonist that blocks β1- and β2-adrenoreceptors, inhibits activation of the p38 MAPK pathway [68], and then down-regulates the expression of proinflammatory cytokines such as interleukin (IL)-1β and IL-8 (Figure 3). Wang et al. also compared the roles of carvedilol and metoprolol for the treatment of viral myocarditis [68] and found that carvedilol improved cardiac contractility and diastolic function, while metoprolol only improved contractile function, indicating that carvedilol protects heart function by mechanisms other than inhibiting β-adrenergic receptors. It may exert a variety of pharmacological effects, such as anti-oxidative stress and myocardial remodeling, but further studies are needed to confirm these effects.

Apart from drug screening, evidence-based medicine and retrospective analysis of cases may also help researchers to identify approved drugs that can be repurposed for EV71 infection, such as carvedilol, which blocks calcium channels and is suitable for the treatment of symptomatic heart failure and essential hypertension. Gong et al. found that carvedilol reduced norepinephrine and epinephrine levels, diastolic and systolic pressure, blood glucose level, heart rate, body temperature, and leukocyte count in children with EV71 HFMD [69], suggesting that carvedilol may alleviate the effects of HFMD.

**Figure 4:**
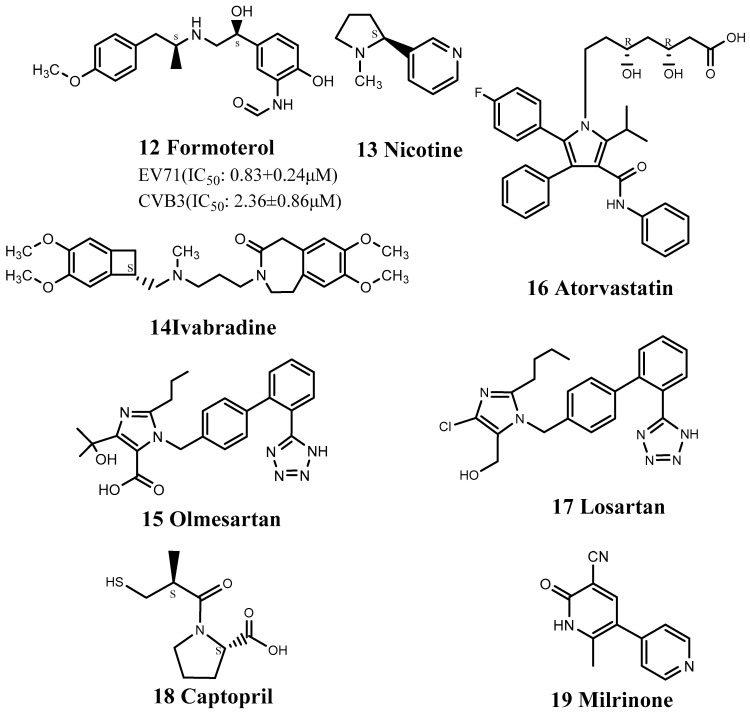
Structures of cardiovascular drugs.

In contrast to carvedilol, formoterol (12) is a β2 receptor agonist that can activate the p38 MAPK pathway and which also inhibited CVB3-induced myocarditis [70] (Figure 4). Rachel et al. found that high concentrations of β2 receptor agonists and inhibitors had no antiviral effects against CVB3 infection, indicating that formoterol did not exert its anti-CVB3 effect by stimulating the β2 receptor. Although the antiviral mechanism of formoterol remains unclear, it acts as a pan-enterovirus inhibitor.

In addition to drugs that act on β-receptors, nicotine (13) may also improve inflammation (Figure 4). Nicotine is an alkaloid found in Solanaceae plants (Solanum) and an α7-nicotinic acetylcholine receptor antagonist, which mainly acts on α-cholinergic receptors. Zhao et al. used nicotine in a mouse model of CVB3 infection and found that the survival rate of BALB/C mice in the nicotine group after 14 days was 80%, compared with only 45% in the drug-free group [71]. Nicotine also significantly reduced myocardial damage and cell infiltration and down-regulated proinflammatory cytokines such as IL-6 and IL-17A. These findings suggest that nicotine can effectively improve myocarditis caused by CVB3 [71].

There are many types of proinflammatory cytokines, and the generation of nitric oxide (NO) is likely to cause myocardial necrosis and contractile dysfunction, suggesting that suppression of NO may reduce the severity of myocarditis. Ivabradine (14) and olmesartan (15) inhibited NO synthesis by inhibiting inducible NO synthase, thereby treating viral myocarditis (Figure 4). Ivabradine (trade name: Corlanor), a selective atrionector-specific If current blocker that slows sinus rhythm, was approved by the FDA in 2015 for the treatment of patients with heart failure through a priority review process. Ivabradine also reduces myocardial damage and down-regulates the expression of proinflammatory cytokines [72]. Olmesartan is an angiotensin II receptor antagonist that can be used as an antihypertensive drug.

The FDA-approved drugs atorvastatin (16) and losartan (17) can also be used as immunomodulators to treat viral myocarditis (Figure 4). Atorvastatin reduced the abnormal expression of tumor necrosis factor-a and IFN-c and restored the expression of connexins such as Cx43 and Cx45 [73], thereby reducing the rate of myocardial necrosis and effectively treating viral myocarditis. Atorvastatin (trade name: Lipitor) is a lipid-lowering drug developed by Pfizer, which is mainly used to treat hypercholesterolemia and coronary heart disease. Losartan can down-regulate the expression of Th17 cells and stimulate Th1 cells to secrete relevant cytokines. Losartan significantly reduced mortality in cytomegalovirus (CMV)-infected mice from 40.0% to 12.5% [74]. Losartan (trade name: Cozaar), as the first angiotensin II receptor antagonist class antihypertensive drug, blocks the key sex hormone angiotensin II to regulate blood pressure.

Captopril (18) is an angiotensin-converting enzyme inhibitor used to treat hypertension and certain types of congestive heart failure (Figure 4). It can reduce myocardial calcification and fibrosis by an unknown mechanism, possibly related to resistance to mitochondrial damage [75]. Milrinone (19) has been used to treat pulmonary edema, as a potentially fatal complication of EV71 infection (Figure 4). Milrinone is an FDA-approved drug that enhances myocardial contractility and directly expands blood vessels. It is used clinically for the treatment of chronic congestive heart failure and intractable heart failure. Although it cannot directly inhibit the production or replication of EV71, milrinone exerts immunomodulatory and anti-inflammatory effects in the management of systemic inflammatory response to severe EV71 infection and has demonstrated good clinical therapeutic efficacy against EV71-related brainstem encephalitis [63].

### 3.4. Repositioning of Nervous System Drugs

**Figure 5:**
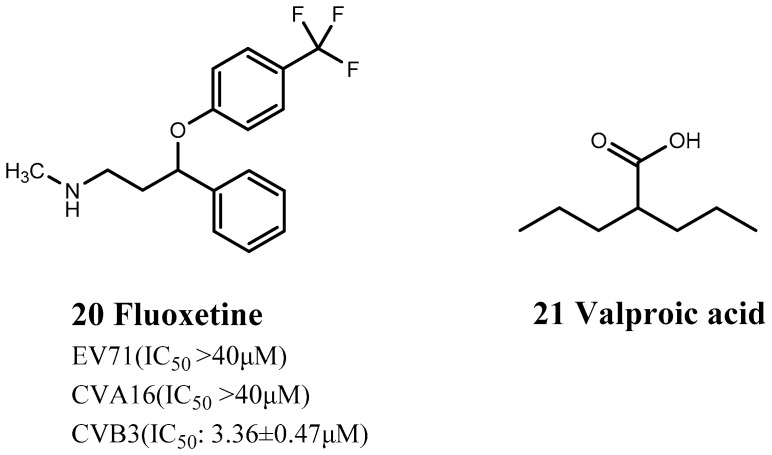
Structures of nervous system drugs.

Bauer et al. discovered that fluoxetine (20) inhibited CVB3 proliferation by inhibiting the non-structural viral protein 2C and reducing the synthesis of CVB3 RNA negative strands [76] (Figure 5). Fluoxetine is a widely used selective serotonin/5-hydroxytryptamine serotonin (5-HT) reuptake inhibitor (SSRI) that selectively inhibits the 5-HT transporter, blocks presynaptic membrane reuptake of 5-HT, and increases the action time of 5-HT, thus producing an antidepressant effect. Ulfert et al. previously identified fluoxetine in independent screens as an inhibitor of EV-B and EV-D members and showed that it acted on viral protein 2C [76]. Bauer et al. demonstrated that the antiviral activity of fluoxetine was stereoselective: it has a chiral center and only the S-enantiomer has antiviral activity, and anti-viral treatment with (S)-fluoxetine alone can thus reduce any potential SSRI-related side effects [76]. This highlights the potential of identifying more specific drug structures for the target disease through structural biology or molecular dynamics simulations, thus reducing some pharmacological effects while improving others during drug repositioning.

Valproic acid (21) is used to treat epilepsy and mania and can act as an immunomodulator by regulating the related functions of immune cells, thereby reducing the severity and mortality of myocarditis (Figure 5). Jin et al. found that valproic acid directly inhibited Th17 cells and upregulated the expression of Treg cells [77]. It also reduced the secretion of IL-17A and IL-10 in CMV-infected mice, which show increased serum and myocardial levels of these cytokines, thereby reducing the severity of myocarditis and increasing survival.

### 3.5. Repositioning of Anticancer Drugs

**Figure 6:**
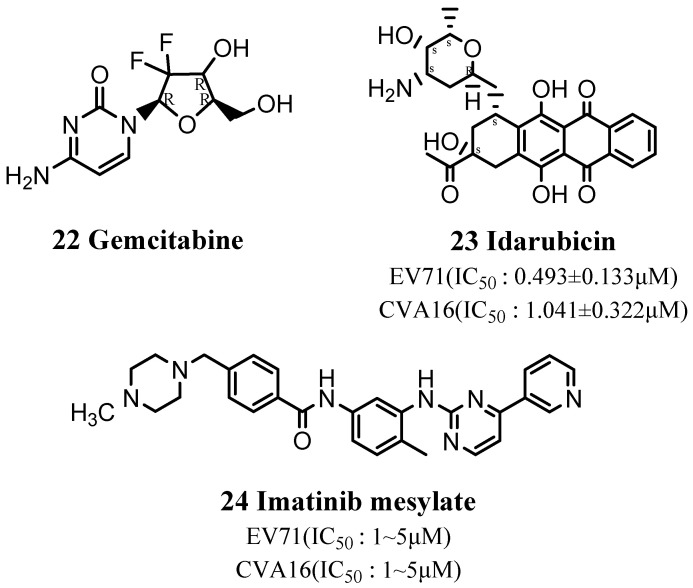
Structures of anticancer drugs.

Gemcitabine (22) is a broad-spectrum viral inhibitor with significant antiviral activity, especially against EV71 and CVB3, and low cytotoxicity [78] (Figure 6). Gemcitabine binds to CVB3 RNA, thus preventing its proliferation and inhibiting replication of the virus. Gemcitabine is a pyrimidine anti-tumor drug. Its main metabolite is incorporated into DNA in the cell and mainly acts on the G1/S phase of the cell cycle, which may explain why gemcitabine binds to viral RNA.

Idarubicin (23) is an anthracycline, cell cycle non-specific anticancer drug. Hou et al. demonstrated that idarubicin also acted as an EV71 inhibitor by preventing the synthesis of EV71 virus protein and RNA, while inhibiting the translation process mediated by its IRES [79] (Figure 6).

Imatinib mesylate (24) is a tyrosine kinase inhibitor that can block one or more protein kinases and which is used clinically to treat chronic myeloid leukemia and malignant gastrointestinal stromal tumors (Figure 6). It reduces cardiac fibrosis by inhibiting activation of platelet-derived growth factor (PDGF) receptors [80]. PDGFα receptors are tyrosine kinase receptors, the activation of which is closely related to the occurrence of CVB3-related myocarditis. PDGF can also activate multiple signaling pathways, including MAPK-regulated signaling pathways, and activation of the p38 MAPK signaling pathway is an important factor promoting the secretion of proinflammatory cytokines.

### 3.6. Repositioning of Antidiabetic and Anti-Obesity Drugs

**Figure 7:**
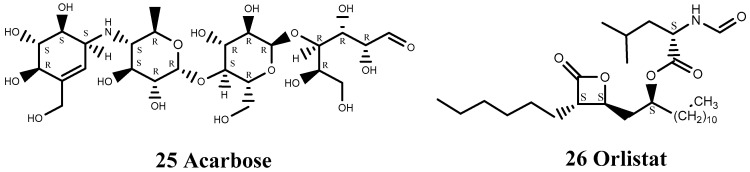
Structures of antidiabetic and anti-obesity drugs.

Acarbose (25) is an α-glucosidase inhibitor that can be used in combination with other oral hypoglycemic drugs or insulin to treat insulin-dependent or non-insulin-dependent diabetes (Figure 7). Experimental research showed that acarbose may prevent EV71 infection by reducing intestinal infection, by blocking the EV71 virus surface receptor binding site, or by inhibiting multiple sugar receptors on the cell surface [81]. Acarbose also showed a good preventive effect at the cellular level.

Orlistat (26) decreases the replication of various viral pathogens by reducing the activity of fatty acid synthase [82] and has demonstrated anti-viral properties, not only against the plus-strand RNA virus CVB3, but also against varicella-zoster virus, indicating that the replication of completely unrelated viruses may depend on the functionality of fatty acid metabolism. Orlistat is an anti-obesity drug that does not act on the central nervous system (Figure 7).

### 3.7. Repositioning of Other Drugs

**Figure 8:**
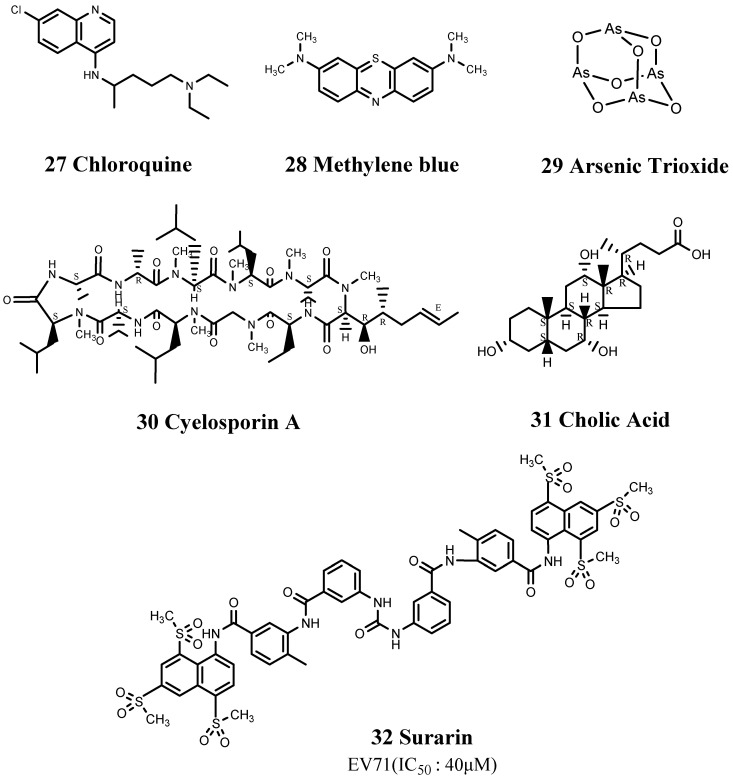
Structures of other drugs.

Zeng et al. also found that the ability of azithromycin (4) to inhibit EV71 infection was comparable to that of chloroquine (27). Chloroquine has been used clinically since 1944, initially to treat malaria, but its use has gradually expanded, and chloroquine was used to treat rheumatoid arthritis in 1951. Shang et al. reported that 1.2 μM of chloroquine resulted in a 104-fold reduction in EV71 RNA synthesis [83], while Shih et al. [84] showed that it blocked the uncoating of EV71 and reduced viral RNA synthesis by >90%. These findings suggest that chloroquine may be an effective EV71 virus inhibitor (Figure 8).

Methylene blue (28) is an aromatic heterocyclic compound that is used as a chemical indicator, dye, biological stain, and drug. It has been shown to destroy EV71 viral proteins and genome in a photodynamically inactive and dose-dependent manner [85] (Figure 8).

Arsenic trioxide (29) was shown by Ylva et al. to reduce the viral load of CVB3 RNA in the pancreas through an unknown mechanism [86] (Figure 8). Arsenic trioxide is an odorless and tasteless creamy white powder and one of the oldest known poisons. It is highly toxic, and close attention should thus be paid to the dosage to prevent unnecessary side effects.

Cyclosporin A (30) is mainly used to prevent rejection of liver, kidney, and heart grafts (Figure 8). It inhibits the opening of mitochondrial permeability transition pores by inhibiting cyclophilin D [87]. ABCC6 mutation is associated with myocardial calcification by increasing the susceptibility of the mitochondria to calcium, etc., resulting in calcification. Cyclosporin A can thus reduce the degree of myocardial calcification by inhibiting mitochondrial permeability transition pores.

In addition, cholic acid (31) 10 μg/mL was shown to reduce production of the viral capsid protein VP1 and inhibit cleavage of the translation initiation factor eIF4G1 [88] (Figure 8). Cholic acid (trade name: Cholbam) is present in bile in cattle, sheep, and pigs and has been approved by the FDA for the treatment of bile acid synthesis disorders and peroxisomal disorders. Moreover, cholic acid inhibited extracellular signal-regulated kinase (ERK) signaling in CVB3-infected HeLa cells [88]. CVB3 infection can cause persistent activation of the host ERK pathway, and inhibition of this pathway using ERK pathway-specific inhibitors, such as PD098059, can significantly reduce the production of related progeny viruses. Cholic acid plays a similar role to PD098059.

Ren et al. selected suramin (32) from 1102 approved drugs. Suramin is used to treat sleeping sickness caused by trypanosomes and has also demonstrated good inhibitory effects against EV71 and CVA16 [89] (Figure 8). Suramin showed no cytotoxicity at concentrations >1 mM, and its original role as a pediatric drug prioritized the possibility of it being repositioned as a treatment for HFMD. Further studies found that structural analogs of suramin, including sulfonated and sulfated compounds, also inhibited the proliferation of EV71, suggesting promising potential and indicating that in addition to the repositioned drug, its structural analogs should also be investigated to identify more effective drug structures. Wang et al. [90] further studied the possible anti-EV71 mechanism of suramin and showed that it inhibited the proliferation of EV71 in both early and late stages of its life cycle and prevented EV71 from attaching to host cells, thereby affecting its entry.

## 4. Conclusions and Perspectives

There are currently no specific antiviral drugs marketed for HFMD, and there is thus an urgent need to develop suitable drugs. Here, we summarized the major outbreaks of HFMD and their causes to outline the etiological and epidemiological features of the disease (Table 1). We also reviewed the progress of drug repositioning for HFMD and summarized the statuses of 32 FDA-approved drugs that have been repositioned for HFMD, including antifungal, antibacterial, cardiovascular, nervous system, anticancer, and other drugs (Table 2).

Among antibacterial drugs, azithromycin (4), which can be used in combination with SPM (5), can inhibit the replication of EV71 and CVA16, thereby exerting significant antiviral effects. It has also been clinically proven to be safe in pregnant women, newborns, and young children. Regarding cardiovascular drugs, lovastatin (7), bosentan (8), and valsartan (9) can inhibit the replication of CVB3 by down-regulating the expression of CAR mRNA and protein. In addition, regarding the roles of carvedilol (11) and metoprolol in the treatment of viral myocarditis, carvedilol can improve cardiac contractility and diastolic function, while metoprolol only improves contractile function, indicating that the cardioprotective action of carvedilol involves other mechanisms in addition to inhibiting β-adrenergic receptors. Formoterol (12) can also inhibit CVB3-induced myocarditis, which seems to indicate an opposite mechanism to carvedilol. Nicotine (13) can effectively improve myocarditis caused by CVB3, reduce myocardial damage and cell infiltration, and down-regulate proinflammatory cytokines. Milrinone (19) has been used to treat the fatal complication of pulmonary edema caused by EV71 infection. Regarding nervous system drugs, valproic acid (21) can act as an immunomodulator by regulating the related functions of immune cells, thereby reducing the severity and mortality of myocarditis. Among anticancer drugs, the main metabolite of gemcitabine (22) is incorporated into DNA in the cell and mainly acts on the G1/S phase. Idarubicin (23) can prevent the synthesis of EV71 virus protein and RNA, while inhibiting the translation process mediated by its IRES. Although most of these drugs are currently in the preclinical research stage, they have shown good antiviral effects at the cellular level, and some have been shown to treat complications in case studies. The drug repositioning strategy allows the drug development time of anti-HFMD drugs to be shortened, and these drug classes and their similar drug structures should thus be prioritized when performing high-throughput screening or chemical structure synthesis.

Currently, almost all available data used in this article to show the potential of repurposed use of known drugs for the treatment of HFMD are from in vitro antiviral activity assays or animal model experiments, except Suramin (32), which is from the Phase I clinical trial. It is expected to provide research clues for drug repositioning for the treatment of HFMD. Due to a lack of clinical trial evidence at this stage, it is not easy to outline a priority list of the drugs discussed. We can, however, roughly classify the drugs discussed basing on their action of mechanisms. According to the past experience in the development of antiviral drugs in the world, we think that drugs that directly target the virus, such as affecting viral RNA synthesis and the binding of viral capsid proteins, have the greatest potential to be repositioned for the treatment of HFMD, including Itraconazole (ITZ) (1), Amphotericin B (3), Azithromycin (4), Minocycline (6), Amiloride (10), Fluoxetine (20), Gemcitabine (22), Idarubicin (23), Acarbose (25), Orlistat (26), Chloroquine (27), Methylene blue (28), Cholic acid (31), and Suramin (32).

Compared with monotherapy, combination drug therapy is also a promising strategy for treating virus infection. The combination uses of pleconaril and ribavirin have been reported against picornavirus, including type 1 diabetes-associated type B coxsackieviruses and foot and mouth disease [91,92]. Efavirenz, a non-nucleoside reverse transcriptase inhibitor, is widely used against HIV. Although the picornavirus does not utilize reverse transcriptase for replication, the combination of pleconaril and efavirenz was found to be superior as compared to pleconaril alone [93]. Amantadine, originally developed as an antiviral drug against influenza infection, also has an antiviral effect on picornaviruses, including hepatitis A virus [94]. Although, currently, there is not so much direct evidence to show that the combination of them could be used to treat HFMD, combination drug therapy is a potential alternative for treating HFMD-related virus infection.

However, although drug repositioning is an effective strategy, it also has some limitations, including the need to be aware of the intellectual property rights of the approved drugs and to consider their original pharmacological effects and limitations. For example, HFMD mainly affects children, while many of the drugs have been approved for adult use, and their feasibility for HFMD thus needs to be carefully assessed. In addition, regarding target selectivity, it is generally difficult for a drug to treat two different diseases simultaneously, and full consideration should thus be given to the affinity of its targets when studying multi-target drugs.

In summary, the drug repositioning strategy has more advantages than disadvantages. It can reduce the drug development process by four to six years, and most approved drugs have adequate post-marketing evaluation reports. However, drug safety is a high priority for the treatment of HFMD, because children are more susceptible to the side effects of drugs than adults. This review summarizes the current status of the drug repositioning strategy for HFMD, with the aim of stimulating further research and improving the hit rate and speed of drug development of antiviral drugs for HFMD.

## Figures and Tables

**Table 1:** Major outbreaks of HFMD and their causes.

Virus	Asia				Others			
EV71	**Area**	**Year**	**Number Infected**		**Area**	**Year**	**Number Infected**	
	Malaysia	1997	41	[9]	California	1974	UNKNOWN	[10]
	China	2008–2012	>7 million	[11]	Bulgarian	1975	700	[12]
	Singapore	2008	29686	[13]	Hungary	2000	UNKNOWN	[14]
	Taiwan	1998	1.5 million	[15]	Austria	2002	12	[16]
	Japan	2010	2900	[17]	Holland	2007	58	[18]
	Vietnam	2005	173	[19]	Denmark	2007	29	[20]
	Russia	2013	UNKNOWN	[21]	Marseille, France	2009	3	[22]
CVB3	Hong Kong	2008	<100	[23]	Poland	1999–2014	<55	[24]
	India	2009–2010	15	[25]				
	Hebei, China	2010–2012	26	[26]				
	Shandong, China	2016	42	[27]				
CVA16	Shanghai, China	2014–2016	144	[28]	Toronto	1957	<100	[29]
					Birmingham	1959	24	[30]
					USA	1959	UNKNOWN	[31]
					Germany	2006	<500	[32]
CVA6	India	2009–2010	<89	[33]	Finland	2008	<50	[34]
	GZ, China	2010–2012	720	[35]	France	2010	25	[36]
	BJ, China	2015	<56	[37]	Spain	2010–2012	<30	[38]
	Thailand	2012	<600	[39]				
CVA10	Japan	1981–1982	18	[40]	USA	1950/2016	UNKNOWN	[40]
	Tajikistan	2004	UNKNOWN	[40]	Germany	2003	UNKNOWN	[40]
	China	2004–2008	>1000	[40]	France	2010	<100	[40]
	Russia	2004–2013	UNKNOWN	[40]	Chad	2006	UNKNOWN	[40]
	Singapore	2008	<100	[40]	Austria	2007	UNKNOWN	[40]
					Spain	2008	UNKNOWN	[40]
					Central African Republic	2008	UNKNOWN	[40]
CVB1	India	2007	UNKNOWN	[41]	USA	2006–2008	235	[42]
	Korea	2008	>104	[43]	Spain	2008	<100	[44]
	Taiwan	2008–2010	>22	[41]				
CVB2	Jiangsu, China	2009	<111	[45]	São Paulo State, Brazil	2004	<10	[46]
	Cambodia	2012	<50	[47]				
	Thailand	2016	<20	[48]				
CVA4	Taiwan	2004–2006	UNKNOWN	[49]	USA	1950	UNKNOWN	[49]
	BJ, China	2011	21	[50]				
	Hangzhou, China	2016	3	[51]				
CVB5	Shandong, China	2005	>54	[52]	Thuringia, Germany	2010	<7	[53]
	Zhejiang, China	2013	<92	[54]	Chiba Prefecture, Japan	2016	3	[55]
CVA9	Gansu, China	2005	<85	[56]	Alberta, Canada	2010	174	[57]
					Mossel Bay, South Africa	Dec 2015–Jan 2016	>26	[58]

**Table 2:** Approved drugs repurposed for HFMD.

Classification.	Drug	Company (Action Date)	Previous Use(s)	Repurposed Use(s)	Clinical Phases
Anti-fungal	Itraconazole (**1**)	SANDOZ (05/28/2004)	deep fungi infection	a broad-spectrum enterovirus inhibitor	Preclinical
	Micafungin (**2**)	FRESENIUS KABI USA (05/17/2019)	candida infection	may target any step in the early viral infection	Preclinical
	Amphotericin B (**3**)	XGEN PHARMS (04/29/1992)	cryptococcus infection	inhibit the production of EV71	Preclinical
Anti-bacterial	Azithromycin (**4**)	OAK PHARMS INC (04/27/2007)	respiratory tract infection	reduce the RNA and protein levels of EV-71	Preclinical
	Spiramycin (**5**)	Odan Laboratories Ltd. (12/31/1957)	Respiratory infection	inhibit virus RNA replication	Preclinical
	Minocycline (**6**)	FOAMIX (10/18/2019)	broad-spectrum antibiotic	suppress cytokine productions and viral protein expressions	Preclinical
Cardiovascular System	Lovastatin (**7**)	COVIS PHARMA BV (06/26/2002)	hypercholesterolemia	reduce CAR mRNA and protein	Preclinical
	Bosentan (**8**)	PAR PHARM INC (04/26/2019)	pulmonary hypertension (PAH)	reduce CAR mRNA	Preclinical
	Valsartan (**9**)	LUPIN (03/30/2015)	anti-hypertension	reduce CAR mRNA	Preclinical
	Amiloride (**10**)	PAR PHARM (01/22/1986)	liver cirrhosis, edema, primary aldosteronism	affect RNA elongation	Preclinical
	Carvedilol (**11**)	TEVA (09/05/2007)	heart failure and essential hypertension	alleviate the dysfunction caused by HFMD	Preclinical
	Formoterol (**12**)	NOVARTIS (09/25/2001)	chronic asthma	a panentervirus inhibitor; inhibit CVB3-induced Myocarditis	Preclinical
	Nicotine (**13**)	DR REDDYS LABS SA (11/27/1991)	improve inflammation	improve myocarditis caused by CVB3	Preclinical
	Ivabradine (**14**)	AMGEN INC (04/15/2015)	heart failure	reduce myocardial damage	Preclinical
	Olmesartan (**15**)	DAIICHI SANKYO (04/25/2002)	antihypertensive	inhibit the synthesis of NO to treat viral myocarditis	Preclinical
	Atorvastatin (**16**)	SANDOZ INC (05/29/2012)	a lipid-lowering drug	reduce myocardial necrosis and treate viral myocarditis	Preclinical
	Losartan (**17**)	MERCK SHARP DOHME (04/14/1995)	antihypertensive	used as immunomodulators to treat viral myocarditis (VMC)	Preclinical
	Captopril (**18**)	MYLAN (02/13/1996)	hypertension and heart failure	reduce myocardial calcification and fibrosis	Preclinical
	Milrinone (**19**)	WEST-WARD PHARMS INT (05/28/2002)	chronic congestive heart failure	Enterovirus 71 Brain Stem Encephalitis	Preclinical
Nervous System	Fluoxetine (**20**)	APNAR PHARMA LP (08/02/2001)	antidepressant	inhibit CVB3 proliferation	Preclinical
	Valproic Acid (**21**)	WOCKHARDT BIO AG (07/01/1986)	epilepsy	improve myocarditis	Preclinical
Anti-cancer	Gemcitabine (**22**)	HOSPIRA (07/25/2011)	a pyrimidine anti-tumor drug	binds to viral RNA	Preclinical
	Idarubicin (**23**)	FRESENIUS KABI USA (08/04/2009)	non-specific anticancer drug	prevent the synthesis of EV71 virus protein and RNA	Preclinical
	Imatinib Mesylate (**24**)	NOVARTIS (04/18/2003)	chronic myeloid leukemia	reduces cardiac fibrosis	Preclinical
Antidiabetic and Anti-obesity	Acarbose (**25**)	WATSON LABS (05/07/2008)	(non)insulin-dependent diabetes	reduce EV71 intestinal infection	Preclinical
	Orlistat (**26**)	CHEPLAPHARM (04/23/1999)	an obesity treatment drug	decrease the replication of different viral pathogens	Preclinical
Others	Chloroquine (**27**)	SANDOZ (11/30/1995)	malaria	block the uncoating of EV71 and reduce viral RNA synthesis	Preclinical
	Methylene blue (**28**)	PROVEPHARM SAS (04/08/2016)	chemical indicator, dye, and drug	destroy EV71’s viral proteins and genome	Preclinical
	Arsenic Trioxide (**29**)	ZYDUS PHARMS (11/13/2018)	one of the oldest poisons	reduce the viral load of CVB3 RNA in the pancreas	Preclinical
	Cyclosporin (**30**)	SUN PHARMA GLOBAL (08/14/2018)	anti-rejection reaction of liver, kidney, and heart transplantation	reduce the degree of myocardial calcification	Preclinical
	Cholic Acid (**31**)	RTRX (03/17/2015)	peroxisomal disorders	reduce the production of the viral capsid protein VP1	Preclinical
	Suramin (**32**)	BAYER	treat sleeping sickness caused by trypanosomes	Inhibit the proliferation of EV71 and CVA16	Phase 1
